# Supplementary material for: Early discharges in severely injured patients by ISS score: exploring injury patterns and coding practices
Source: Eur J Trauma Emerg Surg. 2026 May 13;52(1):165. doi: 10.1007/s00068-026-03167-8 (PMC13171672; doi:10.1007/s00068-026-03167-8)
Supplement: Supplementary file 1 — Supplementary Material 1 [file 68_2026_3167_MOESM1_ESM.docx]

Supplementary Table1. Most common codes among early discharged patients and their association with lack of information.(The codes related to lack of information are highlighted in orange)

|  | Code | Definition based on AIS 2005(2008 update) | N | | Related to lack of information |
| --- | --- | --- | --- | --- | --- |
| 1 | 140652.4 | Subdural hematoma: small to moderate—volume ≤ 50 cc (≤ 25 cc if age ≤ 10); thickness 0.6–1 cm | 290 | | Yes |
| 2 | 450203.3 | Fracture of ≥ 3 ribs [OIS II] | 186 | | Yes |
| 3 | 150200.3 | Base (basilar) fracture: NFS. | 134 | | Yes |
| 4 | 150202.3 | Base fracture: without CSF leak | 107 | | No |
| 5 | 140651.3 | Subdural hematoma: tiny—thickness < 0.6 cm [includes tentorial (subdural) blood on one or both sides]. | 83 | | No |
| 6 | 140682.3 | Pneumocephalus directly related to head trauma | 82 | | No |
| 7 | 140632.4 | Epidural hematoma: small to moderate—volume ≤ 50 cc (≤ 25 cc if age ≤ 10); thickness 0.6–1 cm | 70 | | Yes |
| 8 | 251900.3 | Panfacial fracture | 64 | | No |
| 9 | 140640.4 | Subcortical hemorrhage: small—volume ≤ 30 cc (≤ 15 cc if age ≤ 10); diameter 1–4 cm (≤ 1 cm if age ≤ 10) | 57 | | No |
| 10 | 150206.4 | Base fracture: complex—open with torn, exposed, or missing brain tissue; comminuted; ring; hinge. | 47 | | No |
| 11 | 140604.3 | Cerebrum contusion: single; NFS | 43 | | Yes |
| 12 | 250808.3 | Maxilla fracture: LeFort III | 43 | | No |
| 13 | 150404.3 | Vault fracture: comminuted; compound but dura intact; depressed ≤ 2 cm; displaced | 38 | | No |
| 14 | 140650.3 | Subdural NFS | 27 | | Yes |
| 15 | 140602.3 | Cerebrum contusion NFS [include perilesional edema for size] | 26 | | Yes |
| 16 | 140606.3 | Cerebrum contusion: small; superficial—volume ≤ 30 cc (≤ 15 cc if age ≤ 10); diameter 1–4 cm (1–2 cm if age ≤ 10); midline shift ≤ 5 mm | 25 | | No |
| 17 | 140638.3 | Cerebrum hematoma: intracerebral NFS [include perilesional edema for size] | 24 | | Yes |
| 18 | 442203.4 | Pneumothorax: major—> 50 % collapse of lung documented on X-ray; persistent air leak. | 23 | | No |
| 19 | 140442.4 | Cerebellum: subdural hematoma : small; moderate; ≤30cc or ≤15cc if ≤age 10; 0.6-1cm thick | 22 | Yes | |
| 20 | 650634.3 | Lumbar vertebral body : major compression (> 20% loss of anterior height) | 22 | No | |
