# Supplementary material for: Early discharges in severely injured patients by ISS score: exploring injury patterns and coding practices
Source: Eur J Trauma Emerg Surg. 2026 May 13;52(1):165. doi: 10.1007/s00068-026-03167-8 (PMC13171672; doi:10.1007/s00068-026-03167-8)
Supplement: Supplementary file 2 — Supplementary Material 2 [file 68_2026_3167_MOESM2_ESM.docx]

STROBE Statement—checklist of items that should be included in reports of observational studies

|  | Item No. | Recommendation | Page  No. | Relevant text from manuscript |
| --- | --- | --- | --- | --- |
| **Title and abstract** | 1 | (*a*) Indicate the study’s design with a commonly used term in the title or the abstract | 1 | Early Discharges in Severely Injured Patients by ISS score: Exploring Injury Patterns and Coding Practices |
|  |  | (*b*) Provide in the abstract an informative and balanced summary of what was done and what was found | 2 | **Purpose** This study examines the prevalence of early discharge (ED) among patients classified as severely injured in the Dutch National Trauma Registry (DNTR) and evaluates whether identifiable patient-, injury-, and system-related factors are associated with the occurrence of ED in this population.  **Methods** This retrospective cohort study analyzed DNTR data from 2015 to 2022, focusing on severely injured patients (Injury Severity Score [ISS] ≥ 16). Patients were grouped as early discharge (ED; discharged within 48 hours without in-hospital mortality) or non-ED (NED). Patients’ characteristics were compared using descriptive statistics, and a mixed-effect model identified factors associated with ED.  **Results** From 2015 to 2022, a total of 37,626 severely injured patients were registered including 1,454 (3.9%) ED patients. This proportion increased from 3.2% in 2015 to 4.8% in 2022. ED was more common in younger patients, males, general practitioner (GP) or self-referred cases, those with severe head injury (Abbreviated Injury Scale [AIS] ≥ 3) and fewer registered injury codes. In the mixed-effects model, Younger age (OR up to 2.0), increasing head injury severity (OR: 1.16 per AIS point), less than 3 registered AIS codes (OR: 0.60), referral by a general practitioner (OR 1.68) or self-referral (OR 1.92), and admission year (OR: 1.08 per year) independently predicted early discharge. Subdural hematoma (small–medium) was the most frequent severe injury code among ED patients.  **Conclusion**  The prevalence of early discharge among patients classified as severely injured has increased in the Netherlands over time. Early discharge was more frequently observed in patients with severe head injuries, those who were self- or general practitioner–referred, and varied across regions. These patterns may reflect differences in clinical practice, trauma system organization, or limitations in AIS/ISS-based injury classification rather than a single cause. Greater attention to coding specificity and injury classification may improve the interpretation of registry-based outcome evaluations. |
| Introduction | | | |  |
| Background/rationale | 2 | Explain the scientific background and rationale for the investigation being reported | 3 | Trauma registries collect data on hospitalized patients who have sustained injuries in order to monitor and assess the quality of trauma care. Maintaining a reliable trauma registry with high-quality data is a critical component of a well-developed and inclusive trauma care system. The standardized data from such registries can serve as benchmarks, enabling comparisons across patients, institutions, regions, and countries. Moreover, these data provide insights for new therapeutic options, more efficient care and prevention measures, ultimately reducing morbidity and mortality rates in regions and countries [1, 2].  Accurate and consistent injury classification is crucial for trauma registries to properly profile injuries, ensure the inclusion of eligible cases and make comparisons [3]. Nowadays, The Abbreviated Injury Scale (AIS) is widely used in trauma registries worldwide to classify injuries [4]. Compared to general administrative coding systems such as the International Classification of Diseases (ICD) [5], the AIS offers specific advantages: it is designed specifically for injuries, provides a more detailed injury characterization, and includes a built-in severity measure. Since AIS coding is a standardized system, it theoretically supports reliable benchmarking across trauma care systems globally. However, this assumes that AIS scoring is applied consistently across and within all systems [4].  Despite the widespread adoption of AIS, its accuracy depends on consistent application by trauma registration employees and/or clinicians. On the other hand, the Injury Severity Score (ISS), a widely used summary measure of overall injury severity, is calculated by squaring and summing the three highest AIS scores from different body regions, meaning that ISS is directly derived from AIS scores [2]. Therefore, inconsistencies in AIS scoring can undermine the reliability of registry data, leading to misclassification of injury severity. This, in turn, may affect performance evaluations, clinical outcome assessments, and resource allocation [6]. Over the years, newer versions of AIS have been developed to enhance clarity and coding precision. While the most recent version is AIS 2015, the AIS 2005 update 2008 version is currently used in trauma registries in the Netherlands [4].  Severely injured patients, defined as those with Injury Severity Score (ISS) ≥ 16, represent one of the most critical and resource-intensive groups within trauma care. These patients typically require prolonged monitoring, repeated assessments, and multidisciplinary management, making early discharge clinically unlikely in most cases. Studies report that the average hospital stay for severely injured patients can vary significantly, but commonly is reported to be more than 8 days [7]. This reflects the severity and complexity of injuries in these patients [7, 8].  Given this context, early discharge (ED), defined as discharge directly to home within 48 hours of admission, is relatively uncommon among patients classified as severely injured. While ED may reflect appropriate clinical decision-making in selected low-risk cases, its occurrence in patients with ISS ≥ 16 raises questions about how injury severity is classified and interpreted within registry data. Such patterns may result from several mechanisms, including limited documentation, reduced coding specificity, structural limitations of the AIS/ISS framework, or coding inaccuracies, and may lead to an overestimation of injury severity relative to the clinical course [9]. |
| Objectives | 3 | State specific objectives, including any prespecified hypotheses | 3 | Therefore, this study aims to examine the prevalence of early discharge among patients with ISS ≥ 16 and to evaluate patient-, injury-, and system-related factors associated with its occurrence. Our working hypothesis is that identifiable characteristics, either in injuries or coding, may help explain why early discharge occurs in a subset of patients classified as severely injured. |
| Methods | | | |  |
| Study design | 4 | Present key elements of study design early in the paper | 4 | A retrospective cohort study was conducted using data from the Dutch National Trauma Registry (DNTR |
| Setting | 5 | Describe the setting, locations, and relevant dates, including periods of recruitment, exposure, follow-up, and data collection | 4 | The study population consisted of all severely injured cases, defined as patients with an Injury Severity Score [ISS] ≥ 16 , registered in DNTR from 2015 to 2022. |
| Participants | 6 | (*a*) *Cohort study*—Give the eligibility criteria, and the sources and methods of selection of participants. Describe methods of follow-up  *Case-control study*—Give the eligibility criteria, and the sources and methods of case ascertainment and control selection. Give the rationale for the choice of cases and controls  *Cross-sectional study*—Give the eligibility criteria, and the sources and methods of selection of participants | 4 | This registry is a comprehensive national database from the Netherlands that contains information on all trauma patients that are admitted to an emergency department across the Netherlands within the first 48 hours after trauma. The study population consisted of all severely injured cases, defined as patients with an Injury Severity Score [ISS] ≥ 16 , registered in DNTR from 2015 to 2022. Patients for whom the variable of interest was missing were excluded. For this study we used the injuries coded based on AIS 2005 (2008 update). This study has been approved by the scientific committee of DNTR regarding the use of data and data publication(registration number: LTR25.06)  Severely injured cases were stratified into two groups based on discharge timing and outcome. The Early Discharge (ED) group consists of patients with ISS ≥ 16 who were discharged home within 48 hours after admission to the emergency department and did not experience mortality during hospitalization. This is consistent with previous research defining early discharge among trauma patients [11, 12]. The Non-Early Discharge (NED) group consists of all cases with ISS ≥ 16 that did not meet the criteria for early discharge. Polytrauma was defined based on the Newcastle Definition as having injuries with AIS severities more than 2 in at least 2 body regions [13]. |
|  |  | (*b*) *Cohort study*—For matched studies, give matching criteria and number of exposed and unexposed  *Case-control study*—For matched studies, give matching criteria and the number of controls per case |  |  |
| Variables | 7 | Clearly define all outcomes, exposures, predictors, potential confounders, and effect modifiers. Give diagnostic criteria, if applicable | 4 | The primary outcome of interest was the occurrence of ED, defined as discharge to home within 48 hours of hospital admission. The factors associated with ED in severely injured patients, as well as the prevalence and trends of this phenomenon over the study period were identified as the secondary outcomes.  The following patient and injury-related variables were examined as potential predictors of ED: demographic factors included age (categorized into groups) and sex, incident-associated factors included trauma mechanism, injury type (blunt vs. penetrating trauma), total number of AIS codes per patient, referral source (ambulance, self-referred, referred from another hospital, or referred by general practitioner) and the presence of severe injury in any body region (defined as maximum AIS ≥ 3). Outcome measures involved the Glasgow Outcome Scale (GOS), and 30-day mortality.  The frequently repeated AIS codes with a severity score ≥ 3 among ED cases were also evaluated to find the most common AIS codes among ED cases. |
| Data sources/ measurement | 8* | For each variable of interest, give sources of data and details of methods of assessment (measurement). Describe comparability of assessment methods if there is more than one group | *4* | The primary outcome of interest was the occurrence of ED, defined as discharge to home within 48 hours of hospital admission. The factors associated with ED in severely injured patients, as well as the prevalence and trends of this phenomenon over the study period were identified as the secondary outcomes.  The following patient and injury-related variables were examined as potential predictors of ED: demographic factors included age (categorized into groups) and sex, incident-associated factors included trauma mechanism, injury type (blunt vs. penetrating trauma), total number of AIS codes per patient, referral source (ambulance, self-referred, referred from another hospital, or referred by general practitioner) and the presence of severe injury in any body region (defined as maximum AIS ≥ 3). Outcome measures involved the Glasgow Outcome Scale (GOS), and 30-day mortality.  The frequently repeated AIS codes with a severity score ≥ 3 among ED cases were also evaluated to find the most common AIS codes among ED cases. |
| Bias | 9 | Describe any efforts to address potential sources of bias | 5 | Sensitivity Analysis  To evaluate the robustness of the final mixed-effects logistic regression model, several prespecified sensitivity analyses were performed. These included: (1) applying a stricter definition of early discharge as discharge home within 24 hours, (2) restricting the study population to patients with ISS ≥ 17 to exclude borderline injury severity cases, (3) using an alternative parameterization of head injury severity by modeling maximum head AIS as a binary variable (≥ 3 vs. < 3), and (4) conducting leave-one-region-out analyses to assess the influence of regional case-mix. All sensitivity models were adjusted for the same covariates as the primary analysis. |
| Study size | 10 | Explain how the study size was arrived at | 4,5 | The sample size calculation was not applicable for this study. All severely injured cases, defined as patients with an Injury Severity Score [ISS] ≥ 16 , registered in DNTR from 2015 to 2022 were included in the study. |

Continued on next page

| Quantitative variables | 11 | Explain how quantitative variables were handled in the analyses. If applicable, describe which groupings were chosen and why | 4,5 | Descriptive statistics were used to summarize baseline characteristics. Continuous variables were presented as means (SD) or medians (IQR) according to their distribution. Categorical variables were presented as frequencies and percentages. |
| --- | --- | --- | --- | --- |
| Statistical methods | 12 | (*a*) Describe all statistical methods, including those used to control for confounding | 4,5 | A mixed-effect model was developed to assess variables associated with ED. The model included both fixed effects (such as age, number of AIS codes, maximum head AIS, referral source, and the admission year) and a random effects to account for variations over different geographical regions. We first conducted univariate analyses, and variables with a p-value < 0.10 were considered for inclusion in the multivariable model. Selection was further guided by clinical relevance and prior evidence in the literature. Admission year, which demonstrated a linear association with early discharge, was included as a fixed-effect variable, while geographical region was modeled as a random effect to account for regional variation in practice patterns. Model performance was assessed using the Akaike Information Criterion (AIC) and the area under the receiver operating characteristic curve (AUC). |
|  |  | (*b*) Describe any methods used to examine subgroups and interactions | 4,5 | Comparisons between the ED and NED groups were performed using chi-square tests for categorical variables and t-tests or Mann-Whitney U tests for continuous variables, depending on normality. A p-value of < 0.05 was considered statistically significant for all analyses. All statistical analyses were conducted using R (version 4.4.2). |
|  |  | (*c*) Explain how missing data were addressed |  | - |
|  |  | (*d*) *Cohort study*—If applicable, explain how loss to follow-up was addressed  *Case-control study*—If applicable, explain how matching of cases and controls was addressed  *Cross-sectional study*—If applicable, describe analytical methods taking account of sampling strategy |  | - |
|  |  | (*e*) Describe any sensitivity analyses |  | Sensitivity Analysis  To evaluate the robustness of the final mixed-effects logistic regression model, several prespecified sensitivity analyses were performed. These included: (1) applying a stricter definition of early discharge as discharge home within 24 hours, (2) restricting the study population to patients with ISS ≥ 17 to exclude borderline injury severity cases, (3) using an alternative parameterization of head injury severity by modeling maximum head AIS as a binary variable (≥ 3 vs. < 3), and (4) conducting leave-one-region-out analyses to assess the influence of regional case-mix. All sensitivity models were adjusted for the same covariates as the primary analysis. |
| Results | | | | |
| Participants | 13* | (a) Report numbers of individuals at each stage of study—eg numbers potentially eligible, examined for eligibility, confirmed eligible, included in the study, completing follow-up, and analysed | 5 | A total of 37,626 severely injured cases were registered in DNTR between 2015 to 2022. Out of these patients, 1,454 had a hospital stay of ≤ 48 hours and were directly discharged to their home. Among these 1,454 cases 61 patients met the Newcastle criteria for polytrauma. The prevalence of these ED cases among severely injured cases increased significantly over time from 3.2% in 2015 to 4.8% in 2022 |
|  |  | (b) Give reasons for non-participation at each stage |  | - |
|  |  | (c) Consider use of a flow diagram | 5 | **Fig1. The trend of ED percentage in the severely injured cases between 2015 and 2022. The light blue area shows the 95% confidence interval.** |
| Descriptive data | 14* | (a) Give characteristics of study participants (eg demographic, clinical, social) and information on exposures and potential confounders | 6 | Older adults (> 65 years) were the most prevalent age group in both ED and NED patients, though the proportion was significantly lower among ED patients (30.3% vs. 38.9%). In contrast, children and young adults (0–35 years) collectively made up a larger share of ED patients (31.7% vs. 25.6%, *p* < 0.001). Males were the majority in both groups, with a significantly higher proportion in the ED group (70.3% vs. 65.9%, p < 0.001).  Regarding referral patterns, ambulance referrals were the most common in both groups, though slightly less frequent among ED patients (74.1% vs. 77.9%). GP referrals were more common among ED patients compared to NED patients (12.8% vs. 8.0%), while referrals from other hospitals were less frequent in the ED group (6.3% vs. 10.6%). Additionally, self-referrals were more frequent among ED patients (6.4% vs. 3.0%) (p < 0.001 for overall referral distribution).  Mechanism and type of injury  Traffic accidents were the most common cause of injury in both groups, accounting for 41.9% of ED cases and 40.2% of NED cases. Private incidents were also a leading cause in both groups, with similar proportions (41.1% vs. 40.9%). Suicide attempts were substantially less frequent among ED patients compared to NED patients (1.6% vs. 4.5%). Blunt trauma was the predominant injury type in both groups, with no meaningful difference between ED and NED patients (96.8% vs. 96.7%, p = 0.9).  Injury severity and distribution  The ED group had a significantly lower median ISS compared to the NED group (17 vs. 22, p < 0.001). A greater proportion of ED patients had three or fewer registered AIS codes compared to NED patients (38.0% vs. 29.1%, p < 0.001). The head was the most commonly affected anatomical region within the registered severe injuries (AIS ≥ 3) in both groups, with a significantly higher proportion among ED patients (69.5% vs. 54.0%, p < 0.001). Severe thoracic injuries were notably less prevalent in ED patients compared to NED patients (21.8% vs. 40.0%, p < 0.001), as were abdominal injuries (2.4% vs. 11.2%, p < 0.001 |
|  |  | (b) Indicate number of participants with missing data for each variable of interest |  | - |
|  |  | (c) *Cohort study*—Summarise follow-up time (eg, average and total amount) |  | NA |
| Outcome data | 15* | *Cohort study*—Report numbers of outcome events or summary measures over time | 6,7 | Mild disability was the most prevalent outcome in both groups, with a slightly higher frequency among ED patients (52.0% vs. 44.9%), while severe disability was substantially less common in ED compared to NED patients (2.7% vs. 19.8%). Good recovery was markedly more frequent among ED patients (45.0% vs. 15.3%). No in-hospital deaths were observed in the ED group by definition, whereas 18.9% of NED patients died during hospitalization (p < 0.001).  The 30-day mortality rate was also substantially lower among ED patients compared to NED patients (2.6% vs. 23.7%, p < 0.001). |
|  |  | *Case-control study—*Report numbers in each exposure category, or summary measures of exposure |  | *-* |
|  |  | *Cross-sectional study—*Report numbers of outcome events or summary measures |  | *-* |
| Main results | 16 | (*a*) Give unadjusted estimates and, if applicable, confounder-adjusted estimates and their precision (eg, 95% confidence interval). Make clear which confounders were adjusted for and why they were included | 8,9 | In order to assess the prognostic factors of ED among patients with ISS ≥ 16, a mixed-effects logistic regression model was used. The model showed strong discriminative ability (AUC = 0.90) and an acceptable overall fit (AIC = 15,892.5).  The mixed-effects model identified several significant predictors of early discharge (ED). Compared with patients older than 65 years, all younger age groups showed significantly higher odds of ED, including patients aged 0–17 years (odds ratio [OR] = 1.88, 95% CI: 1.54–2.30, p < 0.001), 18–35 years (OR = 2.00, 95% CI: 1.72–2.33, p < 0.001), 36–50 years (OR = 1.92, 95% CI: 1.62–2.27, p < 0.001), and 51–65 years (OR = 1.56, 95% CI: 1.34–1.82, p < 0.001).  Head injury severity was significantly associated with ED, with increasing maximum head AIS scores linked to higher odds of early discharge (OR = 1.16 per AIS point increase, 95% CI: 1.13–1.20, p < 0.001). In contrast, patients with more than three registered AIS codes had significantly reduced odds of early discharge compared with those with three or fewer codes (OR = 0.60, 95% CI: 0.53–0.67, p < 0.001).  Referral source was also independently associated with ED. Compared with ambulance referrals, patients referred by a general practitioner (OR = 1.68, 95% CI: 1.41–2.00, p < 0.001) and self-referred patients (OR = 1.92, 95% CI: 1.52–2.42, *p* < 0.001) had higher odds of ED, whereas referrals from another hospital were associated with significantly lower odds of ED (OR = 0.54, 95% CI: 0.43–0.68, p < 0.001).  Admission year showed a significant positive association with ED, indicating increasing odds of early discharge over time (OR = 1.08 per year, 95% CI: 1.07–1.09, p < 0.001) (Table 2). |
|  |  | (*b*) Report category boundaries when continuous variables were categorized |  | - |
|  |  | (*c*) If relevant, consider translating estimates of relative risk into absolute risk for a meaningful time period |  | - |

Continued on next page

| Other analyses | 17 | Report other analyses done—eg analyses of subgroups and interactions, and sensitivity analyses | 9 | Sensitivity analyses demonstrated that the findings of the primary model were robust across alternative model specifications. The direction and magnitude of associations for age, head injury severity, number of registered AIS codes, and admission year remained largely unchanged across all analyses. Effect sizes were generally stronger when applying a stricter definition of early discharge, while restriction to ISS ≥ 17 and alternative parameterization of head injury severity yielded results comparable to the baseline model. Leave-one-region-out analyses showed minimal variation in effect estimates, indicating that the results were not driven by any single region. |
| --- | --- | --- | --- | --- |
| Discussion | | | | |
| Key results | 18 | Summarise key results with reference to study objectives | 10-12 | The observed increase in EDs from 2015 to 2022 aligns with the annual DNTR reports, which similarly show a rising proportion of patients with ISS ≥ 16 being discharged within two days in recent years [14]. This trend can partly be explained by possible gradual improvements in trauma care efficiency [10], evolving clinical management protocols, and occasional resource or capacity constraints within hospitals. As our dataset does not include detailed information on hospital policies or system-level changes over time, these explanations cannot be confirmed, but should be considered when interpreting the temporal pattern.  While taking these clinical, organizational, and system-related considerations into account, it nevertheless remains difficult to reconcile direct discharge to home within 48 hours, in good clinical condition, with the expected clinical course of most patients classified as severely injured (ISS ≥ 16) [8]. To explore this discrepancy further, we examined the characteristics associated with early discharge and identified several patterns that may help contextualize these findings. First, ED patients were more often self-referred or referred by a general practitioner, which may reflect differences in triage pathways or injury severity at presentation. Second, early discharges were not evenly distributed across trauma regions, suggesting that regional variation in clinical practice, system organization or coding may influence discharge decisions. Third, a notable proportion of ED cases were assigned AIS codes with limited specificity or incomplete detail, which may contribute to higher ISS scores despite relatively mild clinical presentations. Finally, ED patients were more frequently coded with severe head injuries, a category that can encompass a wide range of clinical scenarios, including injuries that may be safely discharged early under current traumatic brain injury management protocols.  A total of 19.4% of ED (vs. 11% NED) cases were either self-referred or referred by a GP. This is consistent with previous studies suggesting that patients referred by GPs or self-referred typically have less severe injuries and shorter hospitalizations [15, 16]. This is also in line with the significantly lower 30-day mortality rate among ED cases. This difference is notable given that all patients met the ISS ≥ 16 threshold and suggests that a considerable proportion of ED cases represent clinically low-risk scenarios despite being classified as severely injured.  Another important finding was that ED cases were not evenly distributed across trauma regions in the Netherlands, with some regions showing a significantly higher ED/NED ratio than others. This regional variation can be reflective of differences in clinical practice patterns, discharge decision-making, trauma system organization, or different AIS coding practices. We were not able to accurately measure these factors with the available data in the current study. Previous Dutch DNTR-based studies have demonstrated that trauma system processes and organization differ somewhat across regions [17, 18]. Analyses of trauma system performance have shown variation in triage, referral pathways, and admission to level-1 trauma centers, indicating that patients with comparable injury severity may follow different care trajectories depending on region [17]. In addition, the description of DNTR highlights that, despite national coordination and standardized definitions, data collection and registration are organized at the regional level, which may introduce variability in registration practices and coding interpretation [18].  Gunning et al. demonstrated only moderate inter-rater reliability of AIS coding for severe head injuries within a Dutch trauma center (ICC = 0.62), indicating that differences in coding interpretation can occur even within a single institution [6]. Similarly, Maduz et al. reported discrepancies in 16% of reassessed AIS codes, with nearly one-quarter of these cases being misclassified as severely injured [9]. These findings highlight that AIS coding is subject to interpretation and lacks a universally accepted gold standard, which may introduce heterogeneity into registry-based analyses.  In our study, ED cases more frequently involved AIS codes with limited specificity, including “not further specified” (NFS) diagnoses. Such codes reflect uncertainty or incomplete clinical detail rather than clear diagnostic entities. The lack of a universally accepted gold standard for applying AIS allows a degree of interpretation during coding, which may introduce variability across cases and settings [19]. Prior work by Oliphant et al. showed that more complex injuries were more likely to receive NFS codes, potentially due to differences in clinical terminology, documentation quality, or data availability at the time of coding [20]. While the presence of these NFS codes does not imply errors, it underscores how limitations in injury description may contribute to higher ISS values that do not always align with the observed clinical course. Educational programs might particularly benefit from addressing communication challenges between clinical specialists and registration employees to ensure more consistent use of diagnostic terminology. Additionally, ongoing reassessment and validation of coding accuracy would help standardize the process and reduce subjectivity.  Furthermore, ED cases were more likely to be registered with severe head trauma, defined as an AIS ≥ 3 [21], compared to NED patients. This finding does not necessarily indicate inappropriate discharge or miscoding, since some head injury patients may have a clinically mild course despite being classified as severely injured based on ISS. However, it should also be noted that head injury has been reported as the most common cause of discordant AIS codes in a previous AIS coding validation audit [22]. This can be explained by the fact that brain injuries are inherently more difficult to categorize due to their nuanced nature and the reliance on the radiologist's descriptive language and measurement precision in imaging reports. Subtle variations in how a radiologist describes hematoma size or accompanying features could lead to different AIS classifications, increasing the risk of human error and inconsistencies [23]. Van Ditshuizen et al. in their study showed that implementing a structured radiologic template for coding traumatic brain injuries can reduce missed and nonspecific codes, increase consistency across centers, and improve classification of severely injured cases. This approach might be an effective option to enhance coding quality, reduce registration burden, and support more accurate patient stratification, specifically in cases with head injury [24].  In addition, the most frequently recorded AIS code in our dataset was 140652.4, which groups both small and medium subdural hematomas under a single category. While this reflects the structure of the AIS 2005/2008 codebook rather than a coding error, it limits clinical granularity. Hematoma volume has been shown to correlate with mortality in acute traumatic subdural hematoma, with larger volumes associated with worse outcomes [25]. Consequently, combining small and medium hematomas within one AIS category may obscure meaningful clinical differences and contribute to injury severity classifications that do not always align with the observed clinical course. This issue is further compounded by the ISS ≥ 16 threshold, as a single AIS 4 lesion—such as a small or medium subdural hematoma—automatically results in classification as a severely injured patient (ISS ≥ 16), even when the overall clinical presentation may be mild. Taken together, these findings suggest that some early-discharge cases may represent genuinely low-risk clinical scenarios that are nonetheless classified as severe due to limitations in injury categorization. In this context, further refinement of head injury coding or improved specification of AIS categories may enhance the clinical interpretability and predictive accuracy of registry-based severity measures. |
| Limitations | 19 | Discuss limitations of the study, taking into account sources of potential bias or imprecision. Discuss both direction and magnitude of any potential bias | 12,13 | This study should be viewed as an exploratory, proxy analysis aimed at identifying patterns that warrant further investigation rather than establishing causal relationships. Its retrospective design and reliance on registry data introduce potential variation in AIS coding and reporting practices, which may influence ISS calculations. Moreover, the registry lacks detailed clinical information—such as precise imaging characteristics, neurological status, or other contextual factors—that could better explain early discharge decisions. Differences in discharge practices or documentation across regions and over time may also have contributed to the observed patterns but could not be fully evaluated with the available data. These limitations should be considered when interpreting the findings, alongside the strengths of the study, including its nationwide coverage and large sample size. |
| Interpretation | 20 | Give a cautious overall interpretation of results considering objectives, limitations, multiplicity of analyses, results from similar studies, and other relevant evidence | 12 | Early discharge in patients with ISS ≥ 16 is likely influenced by several factors that were not captured in our data, including clinical nuance and organizational decisions. Nevertheless, the patterns observed in this study point to a few actionable scenarios. First, incomplete documentation or use of nonspecific AIS codes may lead to overestimation of injury severity, highlighting the need for targeted training and improved documentation practices. Second, limited code specificity—particularly for head injuries—may reduce alignment between coded severity and clinical course, suggesting that more detailed injury classification or structured radiological reporting could be beneficial. In addition, future sensitivity analyses excluding cases in which ISS = 16 is driven by a single injury may help clarify the extent to which these patients differ from poly-injury cases in terms of clinical risk and hospital course. Finally, even when coding is accurate, some injury patterns may carry a lower true risk than reflected by ISS, which should be considered when using registry data for benchmarking or policy decisions. Future studies comparing findings across trauma registries may help distinguish between inherent limitations of the AIS system and local coding or radiological practices. |
| Generalisability | 21 | Discuss the generalisability (external validity) of the study results |  | - |
| Other information | |  | | |
| Funding | 22 | Give the source of funding and the role of the funders for the present study and, if applicable, for the original study on which the present article is based |  | - |

*Give information separately for cases and controls in case-control studies and, if applicable, for exposed and unexposed groups in cohort and cross-sectional studies.
